# Α Novel Sutureless Pterygium Excision Surgery Using Human-Derived Dehydrated Amniotic Membrane

**DOI:** 10.7759/cureus.23839

**Published:** 2022-04-05

**Authors:** Paraskevi T Xanthopoulou, Mohamed Elanwar, Motasim Alzyadi, Anastasios Lavaris, Nickolaos Kopsachilis

**Affiliations:** 1 Head and Neck Surgery, East Kent Hospitals University NHS Foundation Trust, Canterbury, GBR; 2 Ophthalmology, East Kent Hospitals University NHS Foundation Trust, Canterbury, GBR

**Keywords:** pterygium, amniotic membrane, conjuctival, cornea

## Abstract

There are many surgical techniques available for the management of pterygium. Excision with limbal conjunctival autograft is currently the most popular surgical procedure and has been shown to have low recurrence rate and fewer complications. The fixation of limbal conjunctival autograft is performed with either sutures or fibrin glue. The use of sutures results in longer operating time and intense postoperative irritation and epiphora. We describe a new technique for pterygium excision using a human amniotic membrane-derived dry matrix (Omnigen®; Nottingham, UK: Nu-Vision Biotherapies). The use of Omnigen instead of conjunctival autograft in pterygium surgery is a new promising technique and results in shorter operation time, easier graft fixation, reduction in complications, and less postoperative discomfort. This is a simple technique for pterygium surgery with dry amniotic membrane matrix and without the need for anchoring limbal sutures or an assistant holding the graft whilst it is glued in place.

## Introduction

There are currently many surgical techniques available for the management of pterygium [1]. These include bare sclera resection or pterygium excision combined with conjunctival autografting [2] or amniotic membrane transplantation secured with either sutures or fibrin glue [3]. The challenges with the use of fibrin glue are that it is relatively expensive, is used off-label without a "standardized application procedure," and is associated with graft dehiscence issues [4]. Sutures have been associated with iatrogenic inflammation which may negatively influence the clinical outcome [5-9]. In addition, several adjunctive therapies, including the use of beta irradiation and mitomycin C have been recommended due to their anti-fibrotic and anti-angiogenic properties [10,11].

Excision with limbal conjunctival autograft is currently considered the standard of care surgical procedure. However, the use of amniotic membrane with bare scleral has been shown to have low recurrence rate and fewer complications [12]. The use of Omnigen® (Nottingham, UK: Nu-Vision Biotherapies), a dry human-derived amniotic membrane matrix, preserved through a low-temperature vacuum dehydration process, instead of cryopreserved amniotic membrane or limbal conjunctival, is a new technique with many advantages such as easier fixation of the membrane, shorter operation time, reduction in complications and less postoperative discomfort. There are no studies exploring the use of Omnigen® in the surgical management of pterygium. However, Maqsood et al. have presented a pilot study to demonstrate the efficacy and safety of the use of Omnigen® in the management of persistent epithelial defect of various etiologies with promising results [13]. We present a novel technique to perform pterygium excision without the need for anchoring limbal sutures or fibrin glue to hold the graft.

## Technical report

Topical anesthesia, namely drops of tetracaine hydrochloride 1% and 0.5% oxy-bupivacaine, is used preoperatively. The surgical field is cleaned with povidone-iodine and a sterile drape is put in place. After insertion of a wire speculum, 1% lidocaine plus adrenaline (1:200,000) is injected underneath the conjunctiva, above the pterygium body.

**Figure 1:**
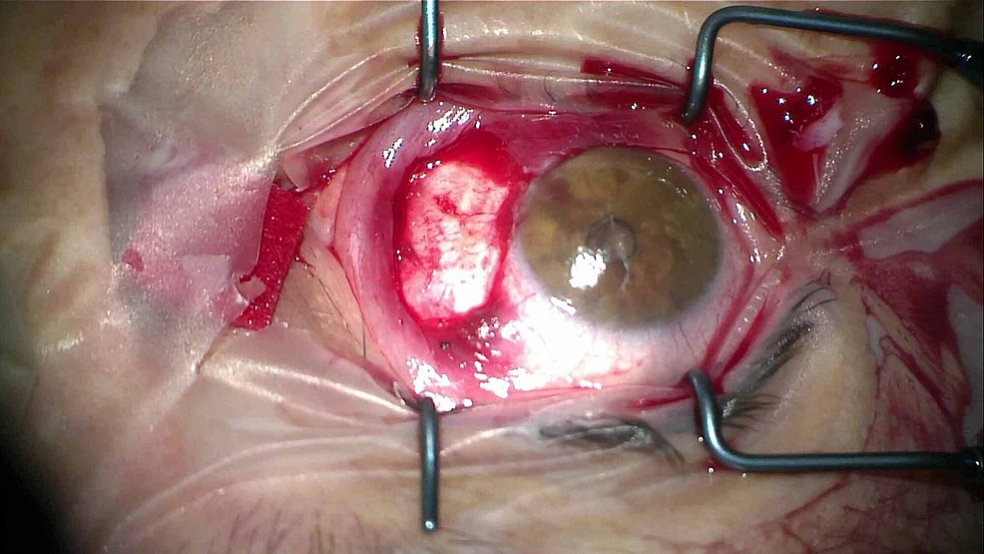
The pterygium and the associated subconjunctival fibrotic tissue were resected down to bare sclera.

The conjunctiva is dissected at the pterygium level, with Vannas scissors. Subsequently, the dissection is continued down to bare sclera, so that the pterygium is exposed, free from the conjunctiva and a conjunctival pocket is created. Pterygium was graded mild (covering less than 1/3 of corneal diameter) based on the corneal progress rate as developed by Nejima et al. [14]. The pterygium head is avulsed from the cornea by grasping it with Colibri forceps and using counter traction with a dry swab (Figure 1). The other end of the pterygium is cut using Wescott scissors, making sure to avoid the underlying rectus muscle. Large hemorrhages are cauterized.

**Figure 2:**
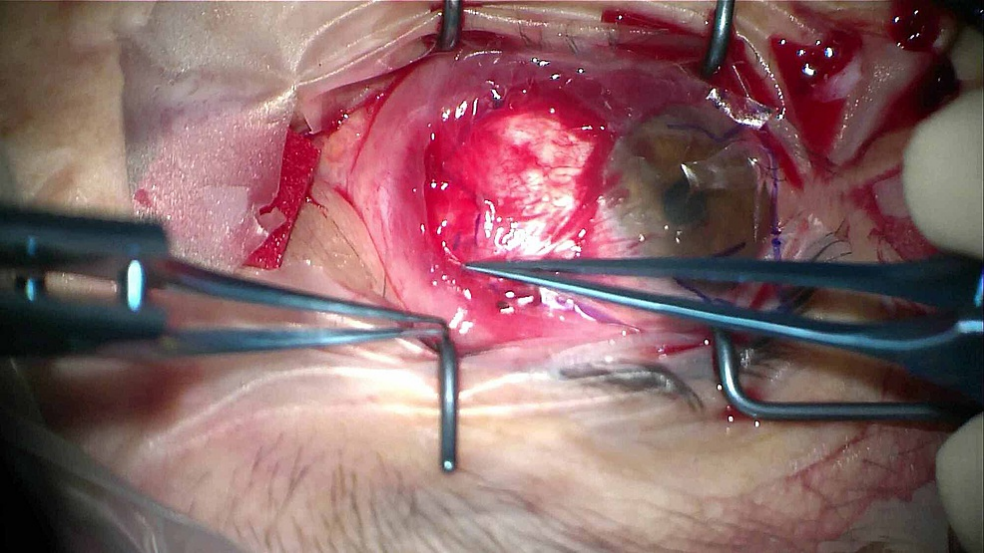
The dry amniotic membrane was oriented to fit the resection site and applied on sclera epithelial-side-up.

**Figure 3:**
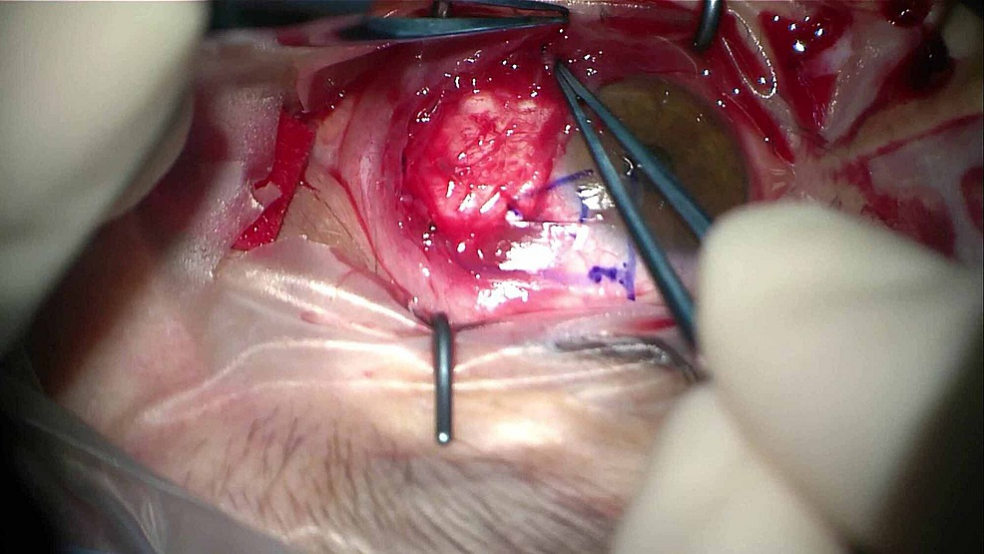
The surrounding conjunctival tissue was mobilized to overlap the edges of the graft.

**Figure 4:**
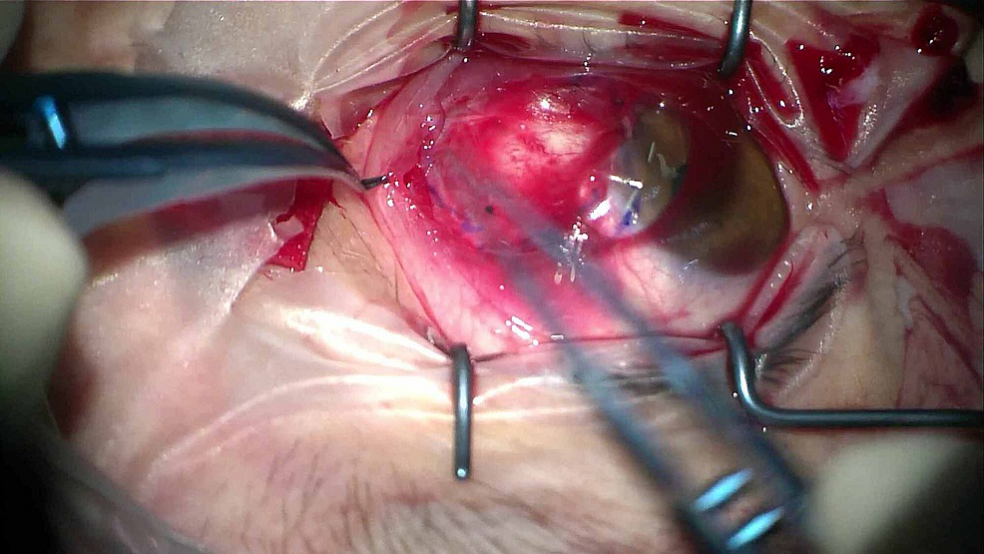
A large bandage contact lens, overlapping the cornea graft conjunctival pocket, was used to ensure stability.

The Omnigen® pre-cut Omnigen amniotic membrane 20 x 20 mm is placed, epithelial-side up, over the bare sclera, after instillation of one drop of balanced saline solution (BSS) (Figure 2). Subsequently, one side of the Omnigen is grasped with non-toothed forceps and positioned below the edges of free conjunctival pocket (Figure 3). After careful positioning of the conjunctival edge over the amniotic membrane, a large (18 mm) therapeutic bandage contact lens (Hydrolens 77; Brackley, UK: Cantor & Nissel Ltd.) is placed on the eye (Figure 4). At the end of the procedure, chloramphenicol 0.5% eye drops are applied with an eye pad. Postoperatively, the patients are treated with chloramphenicol 0.5% eye drops four times daily for two weeks and dexamethasone 0.1% six times a day for two weeks and then four times a day for a month, which is gradually tapered over a period of three months.

Slit lamp examination on days three and seven following surgery showed that the Omnigen® membrane was still in situ. Four-month follow-up showed complete recovery with no evidence of recurrence verified on Pentacam (Oculus Wetzlar, Germany) and anterior segment optical coherence tomography (OCT). Our proposed technique reduced surgical time and also leads to a better postoperative experience for the patient. Additionally, it avoids the need for autograft preparation, anchoring limbal sutures or an assistant to hold the graftin place. 

## Discussion

The standard of care of pterygium surgery is performed with conjunctival autograft [15]; however, emerging studies suggest that with the appropriate surgical technique, the amniotic membrane may deliver safe and effective clinical outcomes and can be less time consuming compared to conjunctival autograft. It also spares the conjunctival for any future surgery [16]. We studied the use of a dry amniotic membrane matrix such as Omnigen® which provides all the nourishing qualities of fresh amnion and overcomes the possible limitations of fresh and cryopreserved amniotic membrane because it can be stored at room temperature (below 25°C). We present a simple 20 minute sutureless and glueless technique of pterygium excision using Omnigen®, a dry amniotic membrane matrix that removes the need for limbal anchoring sutures, or fibrin glue adhesion. 

Omnigen is a unique, stable, easy to access, and easy to use biological matrix that has the potential to be used as an effective regenerative therapy adjunct for a range of ocular surface indications and wide wound care indications. Omnigen is prepared with the Tereo® (Nottingham, UK: Nu-Vision Biotherapies) manufacture process, which preserves the natural structural biochemical composition of fresh amniotic membrane, an element believed to be linked to wound healing performance, thus overcoming the reported limitations associated with conventional cryo- and lyopho-preservation techniques and can retain the natural regenerative properties of fresh amnion in a dry, acellular and stable transplant matrix.

Many surgeons perform pterygium excision using a conjunctival autograft with fibrin glue fixation [17]. In our proposed technique, we place the dry amniotic membrane matrix on bare scleral, undermining the conjunctival frill, and then simply fixating with a bandage contact lens. It appears to be better tolerated than other biological tissues by the patient, not involving reperfusion injury. In addition, this thin avascular biological layer, with immunoregulatory, anti-inflammatory, anti-fibrotic, anti-angiogenic, non-tumorigenic, and wound healing characteristics results in significant pain relief, comfort, and infection control, while facilitating conjunctival regeneration and minimizes scarring. Finally, the addition of a large bandage contact lens ensures that the amniotic membrane graft remains in situ long enough to become reconjunctivalised, thus aiding the healing process.

## Conclusions

In conclusion, this surgical technique demonstrates that pterygium excision with a dry amniotic membrane may represent a safe and effective approach for pterygium excision without the need for anchoring sutures or fibrin glue. Omnigen® not only reduced the operating time needed but also was very well tolerated by the patient. In addition, there was no evidence of recurrence in the four-month follow-up. Further studies are required in order to evaluate long-term outcomes.

## References

[1] Clearfield E, Muthappan V, Wang X, Kuo IC (2016). Conjunctival autograft for pterygium. Cochrane Database Syst Rev.

[2] Mejía LF, Santamaría JP, Cuevas M, Córdoba A, Carvajal SA (2017). Comparison of 4 techniques for limbal-conjunctival autograft fixation in primary pterygium surgery. Eur J Ophthalmol.

[3] Noureddin GS, Yeung SN (2016). The use of dry amniotic membrane in pterygium surgery. Clin Ophthalmol.

[4] Lešin M, Paradžik M, Marin Lovrić J (2018). Cauterisation versus fibrin glue for conjunctival autografting in primary pterygium surgery (CAGE CUP): study protocol of a randomised controlled trial. BMJ Open.

[5] Lee JH, Kang NY (2011). Comparison of fibrin glue and sutures for conjunctival wound closure in strabismus surgery. Korean J Ophthalmol.

[6] Byrne M, Aly A (2019). The surgical suture. Aesthet Surg J.

[7] Demir S, Ortak H, Alim S (2014). Severe conjunctival foreign body reaction caused by polyglactin 910 (vicryl) suture material following strabismus surgery. Turk J Ophthalmol.

[8] Lock AM, Gao R, Naot D, Coleman B, Cornish J, Musson DS (2017). Induction of immune gene expression and inflammatory mediator release by commonly used surgical suture materials: an experimental in vitro study. Patient Saf Surg.

[9] Shahinian L Jr, Brown SI (1977). Postoperative complications with protruding monofilament nylon sutures. Am J Ophthalmol.

[10] Mackenzie FD, Hirst LW (1991). Recurrence rate and complications after beta irradiation for pterygia. Ophthalmology.

[11] Graue-Hernandez EO, Córdoba A, Jimenez-Corona A, Ramirez-Miranda A, Navas A, Serna-Ojeda JC, Mannis MJ (2019). Practice patterns in the management of primary pterygium: a survey study. Cornea.

[12] Rosen R (2018). Amniotic membrane grafts to reduce pterygium recurrence. Cornea.

[13] Maqsood S, Elsawah K, Dhillon N (2021). Management of persistent corneal epithelial defects with human amniotic membrane-derived dry matrix. Clin Ophthalmol.

[14] Nejima R, Masuda A, Minami K, Mori Y, Hasegawa Y, Miyata K (2015). Topographic changes after excision surgery of primary pterygia and the effect of pterygium size on topograpic restoration. Eye Contact Lens.

[15] Ghoz N, Elalfy M, Said D, Dua H (2018). Healing of autologous conjunctival grafts in pterygium surgery. Acta Ophthalmol.

[16] Tasneem AF, Nayak VI, Balakrishna N, Krithika N, Prasad SN (2020). Randomised controlled study of amniotic membrane graft versus conjunctival autograft in primary pterygium excision. IP Int J Ocul Oncol Oculoplasty.

[17] Romano V, Cruciani M, Conti L, Fontana L (2016). Fibrin glue versus sutures for conjunctival autografting in primary pterygium surgery. Cochrane Database Syst Rev.

